# Supplementary material for: Gynae-Oncology Surgeons’ Preparedness to Undertake Colorectal Procedures during Cytoreductive Surgery for Ovarian Cancer: A Cross Sectional Survey
Source: J Clin Med. 2022 Oct 22;11(21):6233. doi: 10.3390/jcm11216233 (PMC9653862; doi:10.3390/jcm11216233)
Supplement: Supplementary file 1 [file jcm-11-06233-s001.zip › jcm-1956493-supplementary.pdf]

File S1: Copy of the online survey sent to members of British Gynaecological Cancer Society (BGCS).

1. Do you work as a consultant Gynae-oncologist?

- Yes
- No

2. Are you GMC accredited with a subspecialty in Gynaecological oncology?

- Yes I am a subspecialist trained in gynae-oncology
- No I have completed the gynae-oncology ATSM
- Other (please specify)

3. Do you work within a tertiary centre or cancer unit? (please add the name of your hospital).

- I work within a tertiary cancer centre setting
- I work within a cancer unit setting
- Other (please specify)

4. What proportion of your operating cases are made up of ovarian cancer cytoreduction (debulking) surgery?

- <20%
- 20–39%
- 40–59%
- 60–79%
- 80–100%

5. With respect to primary surgical cytoreduction (debulking) PSD of advanced ovarian cancer in your department, >50% of stage 3 and 4 will have primary surgical debulking PSD rather than neoadjuvant chemotherapy NACT.

- True
- False

6. Do you have formal training in exenterative surgery including bowel resection with anastomosis or stoma formation?

- Yes I have formal training
- No I do not have formal training

7. Do you have access to colorectal/general surgical support onsite or off site?

- We have access to colorectal/ general surgical support onsite
- We have access to colorectal/ general surgical support on a remote basis

○ Access to emergency oncall team only

8. Do you have a designated colorectal surgeon as support for advanced ovarian cancer surgery if required?

○ We have access to a colorectal surgeon as a formal member of the team who attends cytoreductive surgery as routine.

○ We have access to colorectal support on standby basis should input be required intraoperatively.

○ We rely on the oncall team to attend if required intraoperatively with no formalised arrangement.

9. Do colorectal/general surgery have a formal role in initial MDT pre-operative assessment of ovarian cancer patients for debulking surgery?

○ Yes colorectal/general surgery form part of the MDT assessment of preoperative assessment on a formal basis

○ Colorectal/general surgery are available for advice on preoperative planning

○ Colorectal/general surgery play no role in preoperative planning of cytoreductive surgery patients

10. Thank you thus far. We now hope to further assess the role of colorectal surgery within the setting of ovarian cancer surgery within your department. Please select the link below to continue with this survey, alternatively press to end now

○ Continue survey

○ End survey now

11. Which best describes the role of the colorectal/general surgical team during ovarian debulking surgery within your department?

○ Colorectal/general surgical routinely attend cases with anticipated need for bowel resection

○ Colorectal/general surgical are called to attend if bowel surgery becomes necessary intraoperatively to undertake this

○ Colorectal/general surgical are called to attend to assist with diagnostics and for direct supervision of bowel surgery ultimately undertaken by gynae-oncology

○ Colorectal/general surgical remain remote and the team proceed with bowel resection independently

With regard to specific interventions in the setting of elective surgery with anticipated need for bowel surgery

Please identify the circumstance under which the following would proceed.

Top of Form

12. Small bowel resection with ileostomy?

○ Independently with remote emergency colorectal/general surgical support if required

○ Independently with remote pre planned colorectal/general surgical support if required

Colorectal/general surgical attend theatre for the purpose of direct supervision

Colorectal/general surgical would undertake the procedure

13. Small bowel resection with primary anastomosis?

Independently with remote emergency colorectal/general surgical support if required

Independently with remote pre planned colorectal/general surgical support if required

Colorectal/general surgical attend theatre for the purpose of direct supervision

Colorectal/general surgical would undertake the procedure

14. Right hemicolectomy with no anastomosis (bowel stoma)

Independently with remote emergency colorectal/general surgical support if required

Independently with remote pre planned colorectal/general surgical support if required

Colorectal/general surgical attend theatre for the purpose of direct supervision

Colorectal/general surgical would undertake the procedure

15. Right hemicolectomy with primary anastomosis?

Independently with remote emergency colorectal/general surgical support if required

Independently with remote pre planned colorectal/general surgical support if required

Colorectal/general surgical attend theatre for the purpose of direct supervision

Colorectal/general surgical would undertake the procedure

16. Transverese colectomy with colostomy

Independently with remote emergency colorectal/general surgical support if required

Independently with remote pre planned colorectal/general surgical support if required

Colorectal/general surgical attend theatre for the purpose of direct supervision

Colorectal/general surgical would undertake the procedure

17. Transverse colectomy with primary anastomosis?

Independently with remote emergency colorectal/general surgical support if required

Independently with remote pre planned colorectal/general surgical support if required

○ Colorectal/general surgical attend theatre for the purpose of direct supervision

○ Colorectal/general surgical would undertake the procedure

18. Left hemicolectomy with colostomy?

○ Independently with remote emergency colorectal/general surgical support if required

○ Independently with remote pre planned colorectal/general surgical support if required

○ Colorectal/general surgical attend theatre for the purpose of direct supervision

○ Colorectal/general surgical would undertake the procedure

19. Left hemicolectomy including recto-sigmoid resection with primary anastomosis?

○ Independently with remote emergency colorectal/general surgical support if require

○ Independently with remote pre planned colorectal/general surgical support if required

○ Colorectal/general surgical attend theatre for the purpose of direct supervision

○ Colorectal/general surgical would undertake the procedure

20. Sigmoid colectomy with primary anastomosis and de-functioning ileostomy?

○ Independently with remote emergency colorectal/general surgical support if required

○ Independently with remote pre planned colorectal/general surgical support if required

○ Colorectal/general surgical attend theatre for the purpose of direct supervision

○ Colorectal/general surgical would undertake the procedure

21. Hartmann's procedure?

○ Independently with remote emergency colorectal/general surgical support if required

○ Independently with remote pre planned colorectal/general surgical support if required

○ Colorectal/general surgical attend theatre for the purpose of direct supervision

○ Colorectal/general surgical would undertake the procedure

22. Peritoneal stripping of the rectum?

○ Independently with remote emergency colorectal/general surgical support if required

Independently with remote pre planned colorectal/general surgical support if required

Colorectal/general surgical attend theatre for the purpose of direct supervision

Colorectal/general surgical would undertake the procedure

23. Resection of bowel mesenteric or serosal disease?

Independently with remote emergency colorectal/general surgical support if required

Independently with remote pre planned colorectal/general surgical support if required

Colorectal/general surgical attend theatre for the purpose of direct supervision

Colorectal/general surgical would undertake the procedure

Unanticipated Intervention

Having considered elective surgery with anticipated need for bowel resection, we now consider emergency intervention such as in the instance of unanticipated bowel injury.

Which circumstance best applies to confidence with your team in the following settings?

Top of Form

24. Non full-wall thickness or Serosal bowel injury

No I would wish colorectal to attend from the point of suspected injury

I would feel confident to diagnose but would wish colorectal to attend

I would feel confident to repair but would wish colorectal to attend

I would feel confident to undertake repair independently without the involvement of colorectal surgeons

Other (please specify)

25. Full wall thickness, including mucosa, bowel injury

No I would wish colorectal to attend from the point of suspected injury

I would feel confident to diagnose but would wish colorectal to attend

I would feel confident to repair but would wish colorectal to attend

I would feel confident to undertake repair independently without the involvement of colorectal surgeons

Other (please specify)

26. De-functioning loop colostomy when performed with large bowel repair

No I would wish colorectal to attend from the point of suspected injury

I would feel confident to diagnose but would wish colorectal to attend

I would feel confident to repair but would wish colorectal to attend

I would feel confident to undertake repair independently without the involvement of colorectal surgeons

Other (please specify)

27. Bowel resection

No I would wish colorectal to attend from the point of suspected injury

I would feel confident to diagnose but would wish colorectal to attend

I would feel confident to repair but would wish colorectal to attend

I would feel confident to undertake repair independently without the involvement of colorectal surgeons

Other (please specify)

Bottom of Form

Post-Operative Management

With regard to post-operative management of patient having undergone bowel surgery, either as elective or unanticipated intervention.

Top of Form

28. With regard to post-operative instructions such as establishment of oral intake

Colorectal surgeons are always involved in formulation of postoperative management plan

We often seek advice regarding post-operative management from the colorectal team

Colorectal surgeons are rarely involved in the formulation of postoperative management plan

Other (please specify)

29. With regard to post operative complications such as anastomotic leak or stoma breakdown

Colorectal surgeons are always involved in the diagnosis and management of complications

We would seek colorectal support in the setting of the above complications in most instances

○ We would rarely seek colorectal support in the setting of the above complications

○ Other (please specify)

Bottom of Form
